# 

**Published:** 1892-09

**Authors:** 


### CONTENTS.

Original Articles—The Radical Cure of Hernia, Particularly with Children—By Thos. H. Manley. M. D., New York.....473
The Care of the Patient During the Lying-in-Period—By M. Caldwell, M. D., Waukeshee, Wis.....482
Children and Disease—By G. E. Martin, M. D., Carthage, S. Dekota..486
Compound Fracture of Inferior Maxillary—Treatment of, etc.,—By T. D. McKown, M. D., Chicamauga, Ga.....489
Society Notes—Clinical Society of Maryland.....491
Editorials—Medical Colleges and Medical Education in the South.....514
A Dream.....517
Notice.....518
Selections and Abstracts—The Human Heart..... 490
Iodide of Strontium.....494
Cocaine in Hay-Fever.....498
Papoid in Gastro-Intestinal Disorders.....499
Substitution and Its Attendant Evils.....502
Reply to "Enlarged Prostate A Myth".....504
On the Diagnostic and Prognostic Value of Tendon Reflexes.....505
The Medical and Surgical Register of the United States.....507
Hot Blanket Packs in the Treatment of Fevers.....508
Topical Treatment of Parenchymatous Keratitis and Corneal Opacities with Mercurial Ointment.....509
Treatment of Typhoid Fever.....509
Etiology and Pathology of Dysentery.....510
Appendicitis—Its Diagnosis, Pathology and Treatment.....511
Employment of Hydrastinin in Uterine Hemorrhages.....512
Antidote for Carbolic Acid.....512
The Treatment of Erysipelas.....512
On the Use of Cocaine in Genital Irritation in Men.....513
Book Reviews.....491
A Treatise on Gynecology—Medical and Surgical—By S. Pozzi, M. D. A New Pronouncing Dictionary of Medicine—By J. M. Keating, M. D. and Henry Hamilton, M.D.—Manual of Skin Diseases—By W. A. Hardaway, M. D.—Essentials of Physics—By F. J. Brockway, M. D.—A Practical Manual of Diseases of the Skin—By G. H. Rohe, M. D.—Essentials of Medical Electricity—By D. D. Stuart, M. D., and E. S. Lawrence, M. D.
Prescription Department.....521-522
Special Notes.....519-520

